# Metagenomic Insight: Dietary Thiamine Supplementation Promoted the Growth of Carbohydrate-Associated Microorganisms and Enzymes in the Rumen of Saanen Goats Fed High-Concentrate Diets

**DOI:** 10.3390/microorganisms9030632

**Published:** 2021-03-18

**Authors:** Ying Zhang, Chao Wang, Along Peng, Hao Zhang, Hongrong Wang

**Affiliations:** 1 Laboratory of Metabolic Manipulation of Herbivorous Animal Nutrition, College of Animal Science and Technology, Yangzhou University, Yangzhou 225009, China; zy528729242@163.com (Y.Z.); momei_ziz@163.com (A.P.); 006384@yzu.edu.cn (H.Z.); 2 Joint International Research Laboratory of Agriculture and Agri-Product Safety, The Ministry of Education of China, Yangzhou University, Yangzhou 225009, China; 3 School of Biomedical Sciences, The University of Western Australia, M Block, Queen Elizabeth II Medical Centre, Nedlands, WA 6009, Australia; chao.wang@research.uwa.edu.au

**Keywords:** goats, thiamine, high-concentrate diet, microorganisms and enzymes, metagenomics

## Abstract

Subacute ruminal acidosis (SARA) is often caused by feeding a high-concentrate diet in intensive ruminant production. Although previous studies have shown that dietary thiamine supplementation can effectively increase rumen pH and modify rumen fermentation, the effect of thiamine supplementation on rumen carbohydrate-related microorganisms and enzymes in goats under SARA conditions remain unclear. Therefore, the objective of the present study was to investigate the effects of dietary thiamine supplementation on carbohydrate-associated microorganisms and enzymes in the rumen of Saanen goats fed high-concentrate diets. Nine healthy mid-lactating Saanen goats in parity 1 or 2 were randomly assigned into three treatments: A control diet (CON; concentrate:forage (30:70)), a high-concentrate diet (HC; concentrate:forage (70:30)), and a high-concentrate diet with 200 mg of thiamine/kg of DMI (HCT; concentrate:forage (70:30)). Compared with the HC group, dietary thiamine supplementation improved ruminal microbes associated with fiber, including *Prevotella*, *Fibrobacter*, *Neocallimastix*, and *Piromyces* (*p* < 0.05). In addition, an increase in the relative abundance of enzymes involved in both fiber degradation and starch degradation, such as CBM16, GH3, and GH97, was observed in the HCT treatment. (*p* < 0.05). Thus, thiamine supplementation can improve carbohydrate metabolism by increasing the abundance of the microorganisms and enzymes involved in carbohydrate degradation. In conclusion, this study revealed the relationship between ruminal microbiota and enzymes, and these findings contributed to solving the problems arising from the high-concentrate feeding in ruminant production and to providing a new perspective on ruminant health.

## 1. Introduction

Dietary carbohydrates play an important role in ruminant nutrition and their digestive and physiological functions and most of the carbohydrates ingested are eventually converted into volatile fatty acids (VFAs) by ruminal microorganisms. These VFAs are the main energy precursors for ruminants, and can meet approximately 70% of their caloric requirements [1]. Fibrous materials and starch are two typical carbohydrates in the diet of ruminants, wherein the fiber mainly consists of cellulose, hemicellulose, and lignin. Rumen microorganisms produce various carbohydrate-degrading enzymes, which convert cellulose, hemicellulose, and starch into disaccharides and further convert them into monosaccharides. Carbohydrates in the diet can provide animals with essential nutrients. Therefore, controlling the proportion of dietary concentrate is among the essential factors that maintain a healthy and stable environment for the rumen microorganisms [2]. However, in most intensive animal production facilities, high-concentrate diets fed to animals tend to stimulate the rapid proliferation and growth of lactate-producing microorganisms in the rumen, resulting in the development of subacute rumen acidosis (SARA) [3]. Recent studies showed that feeding a high-concentrate diet can change the structure of rumen microbiota, leading to the enrichment of lactic acid producing bacteria and a reduction in fibrolytic microbes [4]. Moreover, changes in rumen microorganisms reduced fiber utilization efficiency, which resulted in huge losses in animal production [5].

Our previous study found that thiamine (vitamin B1) had a palliated effect on consequences of SARA in goats suffering from a high-concentrate diet [6]. Thiamine supplementation with the feeding of a high-concentrate diet could increase ruminal pH with a concomitant decrease in ruminal lactate concentration, changing ruminal fermentation parameters [6] and promoting ruminal cellulolytic bacteria communities [7]. Studies have found that thiamine plays a pivotal role in carbohydrate metabolism by serving as a cofactor of enzymes, including transketolase, α-ketoglutarate dehydrogenase, pyruvate dehydrogenase, and branch-chain α-keto acid dehydrogenase [8]. For the normal diet of ruminants, almost 90% of thiamine requirements can be microbial synthesized in the rumen and the remaining 10% can be acquired from the diet. However, the SARA condition can lead to inadequate thiamine synthesis and thiamine deficiency in ruminants [9,10]. Thiamine deficiency might be due to the increase in thiamine degradation by thiaminase or the decrease in the microbial thiamine synthesis activity [11]. However, it remains to be clarified whether thiamine supplementation can promote the orderly proliferation of carbohydrate-related microbial microbiota or improve carbohydrate metabolism through carbohydrate-active enzymes (CAZymes).

To date, there have been few studies that focused on the detailed interpretation of thiamine supplementation of carbohydrate-related microbiota of the rumen in cows [12,13]. In this study, metagenomics were used to investigate the effects of thiamine supplementation on the microorganisms related to carbohydrate degradation in the rumen of Saanen goats fed high-concentrate diets. Moreover, to explore the changes in thiamine supplementation due to carbohydrate metabolic enzymes, the carbohydrate-active enzyme (CAZymes) database combined with metagenomics were used to reveal the potential relationship between microbiota and their functions under the action of thiamine, which provided an accurate and systematic reference for demonstrating the protective effect of thiamine on rumen health.

## 2. Materials and Methods

### 2.1. Animals and Experimental Design

All procedures of this study were performed according to the Animal Protection Law based on the Guide for the Care and Use of Laboratory Animals approved on January 4, 2017 by the Ethics Committee of Yangzhou University (SYXK 2016-0019, Yangzhou, China). Animals were slaughtered by the following procedures: The goats were sacrificed by euthanasia, blindfolded with a white cloth, and injected with anesthetic (sumianxin and diazepam). Then, the goats were kept in a deep anesthetic state and sacrificed by vein bleed.

Nine healthy mid-lactating Saanen goats (36.5 ± 1.99 kg BW; 148 ± 3 DMI) in parity 1 or 2 were selected. The goats were randomly assigned into three treatments: A control diet (CON; concentrate 30: forage 70), a high-concentrate diet (HC; concentrate 70: forage 30), and a high-concentrate diet with 200 mg of thiamine/kg of DMI (HCT; concentrate 70: forage 30). The thiamine (thiamine hydrochloride, purity ≥ 99%; Wanrong Science and Technology Development Co. Ltd., Wuhan, China) was mixed with the diet to feed the goats. Details of the ingredient analysis and chemical compositions of the diets are shown in Table 1. Goats were individually housed, fed ad libitum twice a day, and had free access to fresh water. The entire experiment lasted 8 weeks. At the end of the experiment, 4 h after the last feeding, all the goats were slaughtered for sampling.

### 2.2. Sample Collection

Immediately after slaughter, at least 200 mL of rumen contents were sampled from the ruminal ventral sac in a local slaughterhouse on the last day of the trial. The collected rumen digesta was divided into two parts and filtered through four layers of cheesecloth to separate the liquid and solid. The solid phase was washed using sonication (30 s, 50–60 Hz) with an equal amount of sterile saline, then filtered, and centrifuged (10,000× *g* for 1 min). The supernatant after centrifugation was discarded. Repeat these steps once more for a total of three centrifugations. The concentrated microbial suspension was added into the liquid phase. The aliquot was then transferred to liquid nitrogen (N) after adding glycerin as a stabilizer for rapid freezing, and then stored at −80 °C until DNA extraction.

### 2.3. Total DNA Extraction, Library Construction, and Metagenomics Sequencing

Total DNA was extracted from the frozen ruminal samples using a TIANamp Stool DNA Kit (Tiangen, Beijing, China) according to the manufacturer’s protocols. The purity and the quality of the extracted DNA were assessed by electrophoresis and determined spectrophotometrically by measuring the A260/280 (Beckman DU/800; Beckman Coulter, Inc., Fullerton, CA, USA). Qualified DNA samples were randomly broken into approximately 350 bp fragments using Covaris M220 (Gene Company Limited, Woburn, MA, USA) for paired-end library construction and the whole library was prepared using a TruSeq^TM^ DNA Sample Prep Kit (Illumina Inc., San Diego, CA, USA). Adapters were ligated to the blunt-end fragments, which contained the full complement of sequencing primer hybridization sites. Then, the resulting PCR products were purified and quantified on an Agilent Bioanalyzer 2100 system. Sample labeling was performed on a cBot Cluster Generation System using a TruSeq PE Cluster Kit v3-cBot-HS. Finally, paired-end sequencing was performed on an Illumina HiSeq PE150 (Illumina Inc., San Diego, CA, USA) platform according to the manufacturer’s instructions.

### 2.4. Sequence Quality Control and Genome Assembly

The raw data obtained from the Illumina HiSeq sequencing platform were preprocessed to acquire the clean data using Readfq (v8, https://github.com/cjfields/readfq (On 1 November 2018). The following specific reads were removed: (a) The reads that contained low quality bases (quality threshold value ≤ 38) above a certain portion (length of 40 bp); (b) the reads in which the N base had reached a certain percentage (length of 10 bp); (c) the reads that shared an overlap above a certain portion with the adapter (length of 15 bp). In addition, to remove the interference from the host sequence, sequences were aligned to the host sequences using the Bowtie 2.2.4 software with the parameters of -end-to-end, -sensitive, -I 200, and -X 400 (Bowtie 2.2.4, http://bowtiebio.sourceforge.net/bowtie2/index.shtml (On 1 November 2018).

The clean data were assembled and analyzed [6] by the SOAP de novo software (v2.04, http://soap.genomics.org.cn/soapdenovo.html (On 1 November 2018)), then the assembled scaffolds were broken into the Scaftigs without N [14,15,16]. To acquire the unused PE reads, we mapped the clean reads from all samples to Scaftigs with the Bowtie 2.2.4 software using the parameters of -end-to-end, -sensitive, -I 200, and -X 400. The unused reads were mix-assembled based on K-mer = 55. We used Scaftigs with lengths over 500 bp for subsequent analysis. Data are available under BioProject, accession number PRJNA699123.

### 2.5. Gene Prediction and Functional Database Annotations

Open reading frame (ORF) predictions for Scaftigs were produced from mixed assemblies with the MetaGeneMark (v2.10, http://topaz.gatech.edu/GeneMark/ (On 1 November 2018) software. For the predicted open reading frames (ORFs), the CD-HIT [17,18] software (v4.5.8, http://www.bioinformatics.org/cd-hi (On 1 November 2018) was adopted to redundancy and to obtain the unique initial gene catalogue. The clean data were mapped to the gene catalog to acquire the number of reads in each sample with the Bowtie 2.2.4 software [19,20], and the parameters were -end-to-end, -sensitive, -I 200, and -X 400. The final gene catalog was obtained for further analysis.

Unigenes were aligned to the NR database (version 2018-01-02, https://www.ncbi.nlm.nih.gov/ (On 1 November 2018) of NCBI and the CAZymes database (version 201801, http://www.cazy.org/ (On 1 November 2018) using the DIAMOND software (v0.9.9.110) [21]. For the BLAST result of each sequence, the best BLAST hit was used for subsequent analysis [22,23].

### 2.6. Statistical Analysis

The effects of a high-concentrate diet and thiamine supplementation treatment on the relative abundances of microorganisms and all genes encoding carbohydrate-active enzymes were analyzed using ANOVA and a Tukey’s post hoc test with the SPSS software (version 22.0, Chicago, IL, USA) for multiple comparisons. A *p*-value < 0.05 was considered significant. The principal component analysis (PCA) for carbohydrate-active microorganisms and enzymes was constructed and visualized using the R package “FactoMineR” and “Factoextra” (version 4.0.1; https://www.r-project.org (On 1 November 2018)). The spearman correlation analysis was assessed with the SPSS software (version 22.0, Chicago, IL) and visualized in the R package “corrplot” (version 4.0.1; https://www.r-project.org/ (On 1 November 2018)).

## 3. Results

### 3.1. Sequencing Information

**Figure 1:**
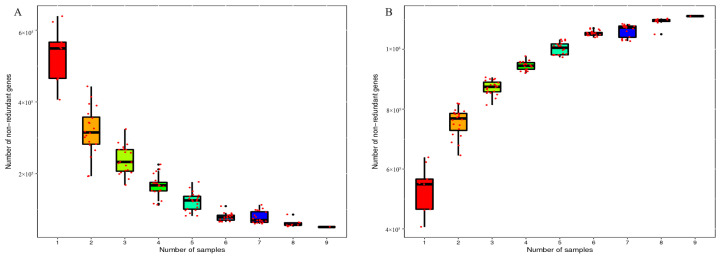
Dilution curve of core-pan gene. The figure (**A**) is the core gene dilution curve; the figure (**B**) is the pan gene dilution curve. The X-axis represents the number of samples taken; the Y-axis represents the number of genes in a sample combination.

Nine metagenomic libraries were constructed. The nine libraries analyzed with the Illumina HiSeq platform gave between 2.5 and 3.4 million sequences in each sample (Table S1). Based on quality control methods, all pollution data were removed, and approximately 6432 M bp per sample were acquired. About 116,748 Scaftigs per sample were assembled with an average length of 1358 bp. A total of 1,153,732 ORFs were obtained, and the average length of each ORFs was 755 bp. Of these, 754,099 ORFs were successfully annotated. The GC content of each sample was between 30% and 55%. The N50 value was between 1185 and 3686 bp for the samples. In the core-pan gene analysis, as shown in Figure 1, the number of non-redundant genes decreased with the increase in the sample number in the core-gene curve, whereas it increased with the increase in the sample number in the pan-gene curve. These results support that the further detailed analysis of metagenome sequencing data was reliable.

The genes annotated in the KEGG database were then matched to the CAZymes database by DIAMOND (https://github.com/bbuchfink/diamond/ (On 1 November 2018)), in which 269 CAZymes were identified. Among these enzymes, four auxiliary activities (AA), 58 carbohydrate-binding modules (CBM), 15 carbohydrate esterases (CE), 120 glycoside hydrolases (GH), 57 glycosyl transferases (GT), and 14 polysaccharide lyases (PL) were identified. These results are shown in Table S2. For the comparison results of each sequence, the best BLAST hit was selected for subsequent analysis.

### 3.2. Effects of Thiamine Supplementation on the Profiles of Carbohydrate-Related Microorganisms and Enzymes

**Figure 2:**
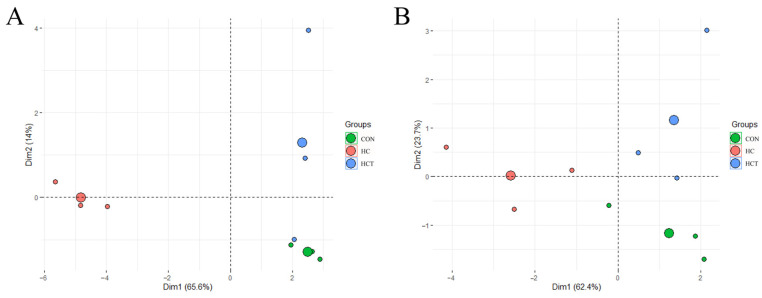
Principal component analysis on the profiles of carbohydrate-related (**A**) microorganisms and (**B**) enzymes in control (CON), high-concentrate diet (HC), and high-concentrate diet supplemented with 200 mg of thiamine/kg of DMI (HCT) treatment.

The principal component analysis (PCA) was used to compare carbohydrate-related microorganisms and enzymes among the three treatments. The PCA analysis of carbohydrate-related microorganisms is shown in Figure 2A, in which axes X and Y were 65.6% and 14.0%, respectively. Similarly, Figure 2B displayed the results obtained from the PCA analysis of enzymes. PCA axes X and Y accounted for 62.4% and 23.7% of the total variation, respectively. The results show that goats fed with high-concentrate diets were considerably different from those in the CON and HCT treatments. On the other hand, the CON treatment was closer to the HCT treatment than to the HC treatment.

**Figure 3:**
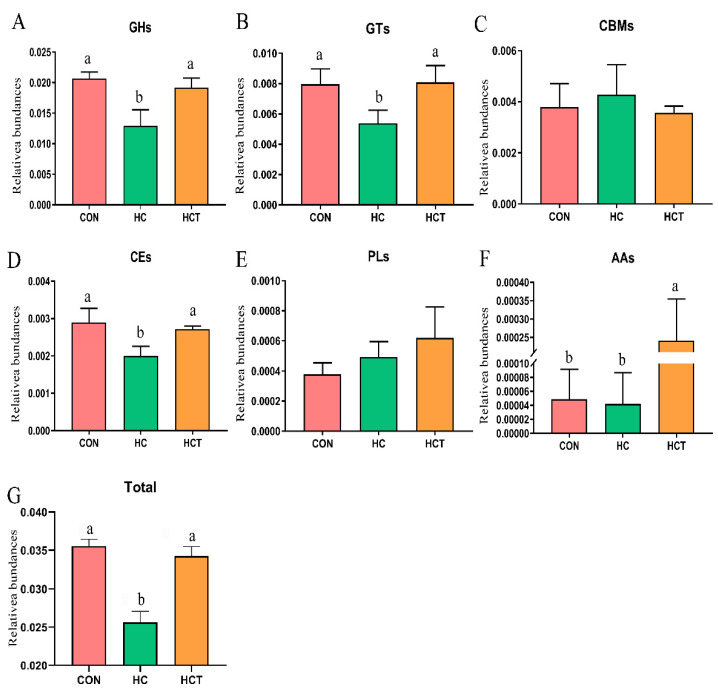
Effects of high-concentrate diet and thiamine supplementation treatment on the relative abundances of carbohydrate-active enzymes. (a−b) Means with different letters differed significantly (*p* < 0.05). CON: Control diet; HC: High-concentrate diet; HCT: High-concentrate diet supplemented with thiamine. GH: Glycoside hydrolases; GT: Glycosyl transferases; CBM: Carbohydrate-binding module; CE: Carbohydrate esterases; PL: Polysaccharide lyases; AA: Auxiliary activity; Total: Sum of relative abundance of GH, GT, CBM, CE, PL, and AA.

Further differential analysis of the data revealed the effects of the three treatments on CAZymes. The results were shown in Figure 3. A lower relative abundance of glycoside hydrolases (GH), glycosyl transferases (GT), carbohydrate esterases (CE), and total CAZymes were found in the goats fed with high-concentrate diets when compared with those on the CON treatment (*p* < 0.05). However, these changes caused by high-concentrate diets were reversed by the HCT treatment and increased the relative abundance of auxiliary activity (AA; *p* < 0.05). In addition, there were no significant differences (*p* > 0.05) in the relative abundance of the other CAZymes, including the carbohydrate-binding modules (CBM) and polysaccharide lyases (PL) among the three dietary treatments.

### 3.3. Effects of Thiamine Supplementation on Carbohydrate-Related Microorganisms Related Fiber and Starch Degradation

**Figure 4:**
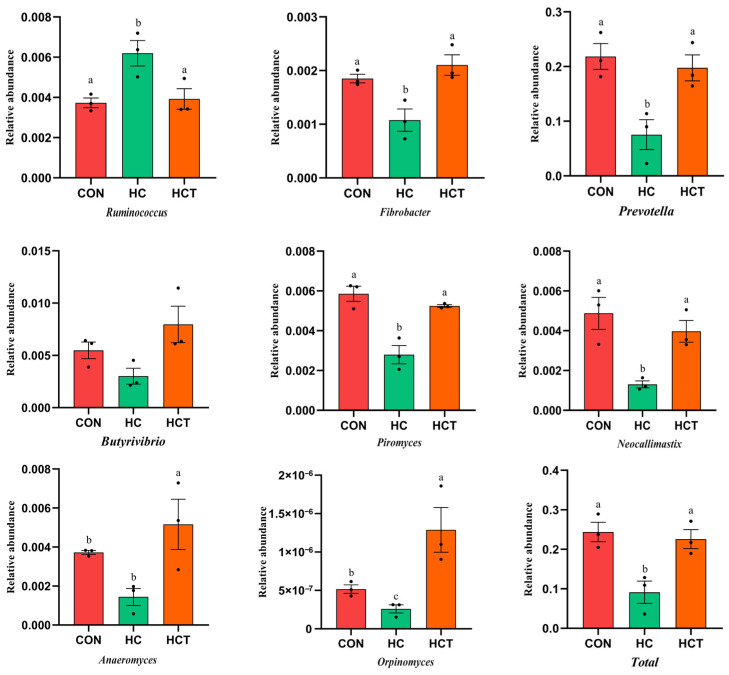
Effects of high-concentrate diet and thiamine supplementation treatment on the relative abundance of fiber-degrading microorganism. (a–c) Means with different letters differed significantly (*p* < 0.05); the error bars represent the standard error of the mean. CON: Control diet; HC: High-concentrate die; HCT: High-concentrate diet supplemented with thiamine.

**Figure 5:**
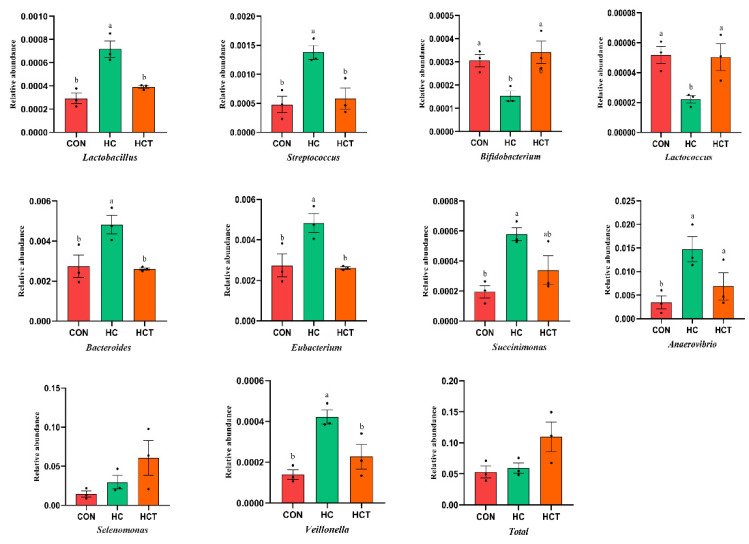
Effects of high-concentrate diet and thiamine supplementation treatment on the relative abundance of starch-degrading microorganism. (a–c) Means with different letters differed significantly (*p* < 0.05). CON: Control diet; HC: High-concentrate diet; HCT: High-concentrate diet supplemented with thiamine.

In order to analyze the microorganisms related to carbohydrate digestion, we filtered the unrelated microorganisms, found the genus level of the related microbiota, and analyzed the differences according to the previous relevant reports [24,25,26]. As shown in Figure 4, compared with the CON treatment, the relative abundance of the fiber-degrading microorganisms (*Fibrobacter*, *Prevotella*, *Piromyces*, *Neocallimastix*, and *Orpinomyces*) was significantly decreased in the HC group (*p* < 0.05). The HCT treatment significantly increased the relative abundance of *Fibrobacter*, *Prevotella*, *Piromyces*, *Neocallimastix*, *Anaerpmyces*, and *Orpinomyces*, but significantly decreased the relative abundance of *Ruminococcus* compared to the CON treatment (*p* < 0.05). Moreover, the total relative abundance of the fiber-degrading microorganisms was increased by the HC treatment. Similarly, starch degrading bacteria were affected differently by the diet, as shown in Figure 5. The relative abundance of *Lactobacillus*, *Streptococcus*, *Bacteroides*, *Eubacterium*, *Succinimonas*, *Anaerovibrio*, and *Veillonella* in the HC treatment were higher than that in the CON and HCT treatments (*p* < 0.05), whereas *Lactobacillus*, *Streptococcus*, *Bacteroides*, *Eubacterium*, *Succinimonas,* and *Veillonella* were similar (*p* > 0.05) between the CON and HCT treatments. The HC treatment reduced the relative abundance of *Bifidobacterium* and *Lactococcus* compared to the CON treatment, but the HCT treatment conversely increased the relative abundance of *Bifidobacterium* and *Lactococcus* compared to the HC treatment (*p* < 0.05). In addition, compared with the HC treatment, the total relative abundance of starch-degrading microorganisms was increased by the HCT treatment (*p <* 0.05).

### 3.4. Effects of Thiamine Supplementation on Fiber-Degrading Enzymes and Starch Degrading Enzymes

In this paper, we studied the effects of dietary treatments on the relative abundance of genes encoding carbohydrate-degrading enzymes based on the annotation of the CAZymes database. The results are presented in Table 2 and Table 3, respectively. Moreover, to gain a deeper understanding of the roles and functions of these enzymes in the different treatments of diets, their main activities are also summarized in Table 2 and Table 3.

Compared with the CON treatment, the relative abundance of four enzymes (CBM6, GH3, GH51, and GH148) was decreased in the HC treatment, which exhibited cellulose-binding and β-glucosidase, endoglucanase, and β-1,3-glucanase activity, respectively (*p* < 0.05). However, the relative abundance of GH9 as cellulases increased with the HC treatment inversely (*p* < 0.05). Meanwhile, GH2, GT2, GH35, GH5, GH67, and CE1 were classified as hemicellulases and exhibited β-galactosidase, β-galactosidase, β-galactosidase, chitosanase, α-glucuronidase, and acetyl xyla esterase activity, respectively, and their relative abundance decreased in the HC treatment (*p* < 0.05). In contrast, the relative abundance of most of the enzymes described above increased in the HCT treatment except for GH35, GH67, and CE1 (*p* < 0.05). Overall, the total relative abundance of fiber-degrading enzymes including the cellulose and hemicellulose-degrading activity, decreased in the HC treatment but increased in the HCT treatment (*p* < 0.05).

In terms of starch-degrading enzymes, the HC treatment decreased (*p* < 0.05) the relative abundance of GH97 and GH13 (which exhibited the activity of glucoamylase and α-amylase, respectively) more than CON or HCT treatment did. The relative abundance of CBM41 in the HC treatment was decreased but not significantly (*p* > 0.05), while that in the HCT treatment was significantly increased (*p* < 0.05). In general, the HC treatment decreased the total relative abundance of starch-degrading enzymes (*p* < 0.05) and the changes in the total relative abundance were inversed by thiamine supplementation (*p* < 0.05).

### 3.5. Relationships between CAZymes and Animal Performance

**Figure 6:**
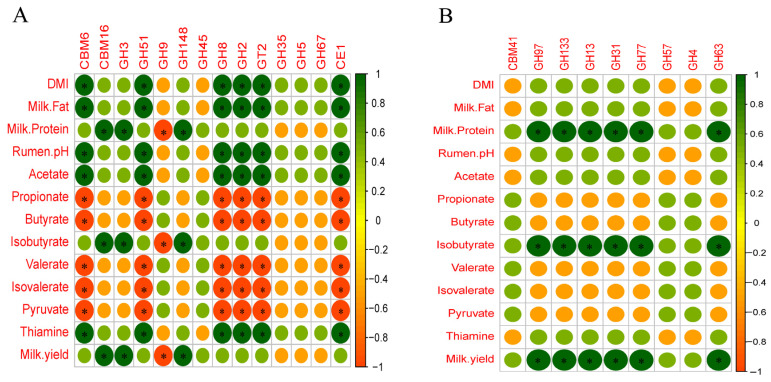
The Spearman correlation analysis between (**A**) fiber-degrading and (**B**) starch-degrading enzymes and animal performance. Green represents positive correlations; red represents negative correlations. “*” Means significant correlations with |*r*| > 0.5 and *p* < 0.05.

Our previous study reported the effect of thiamine on animal performance and rumen fermentation parameters, and these data are shown in Tables S3 and S4 [6]. Due to the close relationship between carbohydrate enzymes and animal production performance, their association may help us better understand their internal relationship. Hence, the Spearman correlation analysis was conducted between CAZymes and ruminal pH, VFAs, DMI, milk quality, milk yield, and thiamine content. The results of the network analysis showed that there were complex associations between carbohydrate enzymes and animal performance. The correlation analysis between fiber-degrading enzymes and animal performance was presented in Figure 6A, in which the relative abundance of CBM6, GH51, GH8, GH2, GT2, and CE1 was positively correlated with DMI, milk fat, ruminal pH, acetate, and thiamine (r > 0.5, *p* < 0.05), while they had negative correlations with propionate, butyrate, valerate, isovalerate, and pyruvate proportions (r < −0.5, *p* < 0.05). The relative abundance of CBM16, GH3, and GH148 was positively correlated with milk protein, milk yield, and isobutyrate (r > 0.5, *p* < 0.05). The relative abundance of GH9 was negatively correlated with milk protein, milk yield, isobutyrate (r < −0.5, *p* < 0.05). As shown in Figure 6B, for starch-degrading enzymes, the relative abundance of GH97, GH133, GH13, GH31, GT3, GH77, and GH63 was positively correlated with milk protein, milk yield, isobutyrate (r > 0.5, *p* < 0.05).

## 4. Discussion

### 4.1. Effects of Thiamine Supplementation on Fiber-Degrading Microorganisms and Enzymes

The rumen is a vast digestive system capable of digesting carbohydrates that are easy or not easy to degrade, including three plant polysaccharides: Fiber, pectin, and starch [27]. Fibrous carbohydrates are indigestible to most animals but for ruminants can be hydrolyzed and fermented by a range of microorganisms in the rumen, which catalyze the degradation of fiber. Fiber degradation requires the synergistic action of a series of enzymes, all of which are produced by rumen microorganisms [24]. Fiber-degrading bacteria and many anaerobic fungi in the rumen have the ability to produce multi-component fiber-degrading enzymes [28]. It is well known that *Prevotella* have the potential to utilize a variety of polysaccharides and are considered to be an important contributor to the degradation of xylan in the rumen. *Ruminococcus* and *Fibrobacter* are both important fiber degrading bacteria in the rumen, among which *Ruminococcus albus* and *Fibrobacter succinogenes* can be commonly observed [29,30,31]. The relative abundance of *Ruminococcus* was increased significantly by the HC treatment, while that of *Fibrobacter* significantly decreased, possibly since *Ruminococcus* can also metabolize other carbon sources such as hexoses and pentoses. Fungi have been reported to be the only known rumen microorganisms with exo-acting cellulase activity [32]. In anaerobic fungi, changes in the relative abundance of genera *Neocallimastix*, *Piromyces*, *Anaeromyces*, and *Orpinomyces* were detected in the HC treatment. Rumen pH drops below 6.5 under the circumstance of a high-concentrate diet fed to animals [33]. However, these microorganisms, including *Fibrobacter*, *Ruminococcus,* and anaerobic fungi were sensitive to mildly acidic pH, and, as such, these cellulolytic microorganisms were unable to fill the “acidic niche” and failed to grow in the same way as on a normal diet. In addition, the diets contain more grain and less fiber in the HC treatment and provide a small amount of nutrients for the fungi, which is an important producer of fiber-degrading enzymes. In this study, the HC treatment significantly decreased the relative abundance of fiber-degrading bacteria and the total relative abundance of fiber-degrading enzymes compared with the CON treatment. Additionally, a decrease in the relative abundance of enzymes involved in fiber degradation in the HC treatment was observed in cows [34]. This result can be explained by the fact that many fiber-degrading bacteria in the rumen are sensitive to low rumen pH and substrate preference, and, thus, they decreased in abundance.

Interestingly, thiamine supplementation significantly increased the relative abundance of fiber-degrading microorganisms and enzymes inversely. We found that thiamine supplementation significantly increases rumen pH and provides a favorable environment for bacteria and fungi, thus promoting their growth in the rumen. In accordance with previous studies, the present results demonstrated that the HCT treatment can increase the relative abundance of *Fibrobacter*, *Prevotella*, *Piromyces*, *Neocallimastix*, *Anaeromyces,* and *Orpinomyces*, which are associated with the degradation of fiber and secreted fiber-degrading enzymes [35,36]. Moreover, it has been reported that thiamine supplementation significantly increases the relative abundance of rumen fungi, including *Ascomycota*, *Basidiomycota*, and *Blastocladiomycota* [12]. Therefore, the increased abundance of bacteria and fungi associated with fiber degradation in the rumen may contribute to the increase in fiber-degrading enzymes.

Research shows that interactions between bacteria and fungi in the rumen, and the maximum fiber degradation ability can be obtained through horizontal gene transfer [37,38]. Interestingly, thiamine was detected to induce a significant increase in fungal growth in the fungi and bacteria co-culture system, and thus the abundance of fiber-degrading enzymes directly increased [39].

In general pathways, carbohydrates are converted first into monosaccharides and then to pyruvate by rumen microbes when ruminants consume high-concentrate diets. Pyruvate, as an important intermediate, plays a large role in many rumen reactions [34]. As the cofactor of pyruvate formate-lyase (PFL) and pyruvate, ferredoxin oxidoreductase (PFO) and thiamine accelerate the conversion rate of pyruvate to acetyl-CoA, which form more ATP to support the metabolism and proliferation of ruminal bacteria and fungi, and also promotes the degradation efficiency of fiber [40].

According to our previous studies, as an essential coenzyme of pyruvate dehydrogenase (PDH), thiamine also can improve the activity of PDH, which accelerates pyruvate flow into the tricarboxylic acid cycle (TCA) and inhibits the flow of pyruvate to lactate and increases the flow of pyruvate to acetate. Consequently, the accumulation of lactate in the rumen is reduced, and the production of acetic acid is increased [6]. Overall, thiamine supplementation also enhanced the total abundance of fiber-degrading enzymes and many individual enzymes including GH2 (β-galactosidase), GH51 (endoglucanase), and CBM6 (binding to cellulose). The results of the present study clearly show that GH2 is positively correlated with rumen acetate concentration, and GH2 and GH51 are positively correlated with milk fat.

### 4.2. Effects of Thiamine Supplementation on Starch-Degrading Microorganisms and Enzymes

In modern intensive production practices, ruminants are frequently fed a high-grain diet, which is high in starch and low in fiber, leading to exposure to the risk of subacute acidosis [41]. In this context, our research focuses on the metabolism of starch in the rumen. In addition to fiber degradation, rumen microorganisms play an important role in starch digestion [42]. We found that the starch-related decomposers were affected differently by high-concentrate diets, which decreased the relative abundance of *Bifidobacterium* and *Bacteroides* and increased the relative abundance of *Succinimonas*, *Anaerovibrio*, *Veillonella*, and *Lactobacillus*. *Bifidobacterium* is a Gram-positive bacterium and has a good ability to degrade starch that can degrade starch into lactic acid and acetic acid [26,43]. *Bacteroides*, as a starch decomposer, was also commonly observed in the rumen. We found that the abundance of *Bacteroides* can be reduced by high-concentrate diets. This suggests that different starch-degrading bacteria have different adaptations and preferences to high-starch diets. In the early stage when ruminants were fed a high-grain diet, and starch-degrading bacteria in the rumen were multiplied in large quantities, starch and other readily fermentable carbohydrates were rapidly fermented into VFAs in the rumen, resulting the decrease in rumen pH [44]. However, the rapid fermentation process failed to constantly continue due to the accumulation of VFA and lactate. Moreover, an acidic microenvironment leads to the inhibition of the bacteria that produce starch-degrading enzymes including *Streptococcus* and *Bifidobacterium* [45,46]. In addition, previous studies have reported that the starch-degrading enzyme is highly adaptable to various pH levels, from 2.0 to 10.5 [42]. These points may explain why there is no increase in total abundance of starch-degrading enzymes in the HC treatment.

Collectively, our results show that thiamine supplementation significantly increased the relative abundances of *Bifidobacterium*, *Bacteroides,* and *Anaerovibrio* and the total relative abundances of starch-degrading enzymes compared with those of the HC treatment. In this study, we found that high-concentrate diets decreased the relative abundance of starch-degrading enzymes, including GH97 and GH13, which is consistent with previous studies [34,47]. Moreover, thiamine supplementation significantly decreased rumen VFAs content and increased ruminal pH, which improved the environmental conditions for starch-degrading microorganisms and promoted their relative abundances. In addition, while promoting fibrinolytic microorganisms, thiamine supplementation catalyzed pyruvate into acetyl-CoA in starch-degrading bacteria, and consequently increased the starch-degrading enzyme profile.

Non-catalytic carbohydrate binding modules (CBMs) bind carbohydrates, and they have been assigned several global functions in CAZyme biology, including coordinated glycan recognition, general substrate adherence, and structure–function contributions to the catalytic site [48]. Therefore, CBMs enable the potentiation and optimization of enzymatic hydrolysis by maintaining the catalytic domain near the substrate, thus helping these enzymes to digest carbohydrates such as starch more efficiently [49]. Moreover, thiamine supplementation reversed the reduced relative abundance of CBM41 in the rumen in the HC treatment and improved the ability of the enzyme to degrade starch.

## 5. Conclusions

In this study, metagenomic sequencing was used to further explore the effects of dietary thiamine supplementation on the relief of high-concentrate diets in Saanen dairy goats from the perspective of carbohydrate-related bacteria and enzymes. We found that thiamine supplementation has the potential to alleviate SARA, improving the abundance of microorganisms associated with carbohydrate-degradation and stabilizing the rumen microbial community. Moreover, we found that the abundance of carbohydrate enzymes was related to the changes in these microorganisms, confirming the close relationship between microorganisms and enzymes. This study provides reference information for resolving the issues caused by feeding ruminants high-concentrate diets in production.

## Figures and Tables

**Table 1:** Ingredients and nutritional composition of diets ^1^ offered to lactating Saanen goats.

Item	CON	HC	HCT
Ingredient (% of DM)			
Chinese wildrye hay	70.00	30.00	30.00
Corn grain	14.00	58.90	58.90
Soybean meal	13.00	8.45	8.45
Calcium hydrophosphate	1.42	0.53	0.53
Limestone	0.58	1.12	1.12
Salt	0.50	0.50	0.50
Premix ^2^	0.50	0.50	0.50
Nutrient composition			
ME (MJ/kg of DM)	8.81	11.72	11.72
CP (% of DM)	10.81	10.79	10.79
NDF (% of DM)	44.28	26.71	26.71
ADF (% of DM)	23.89	13.27	13.27
Starch (% of DM)	11.50	48.38	48.38
Calcium (% of DM)	0.81	0.78	0.78
Phosphorus (% of DM)	0.47	0.42	0.42
Thiamine (mg/kg of DM)	1.20	1.90	201.90

^1^ CON: Control; HC: High-concentrate diet; HCT: High-concentrate diet supplemented with 200 mg of thiamine/kg of DMI. ^2^ Premix consisted of the following ingredients per kilogram of diet: 6.00 × 10^3^ IU of vitamin A, 3.0 × 10^3^ IU of vitamin D, 82.0 mg of vitamin E, 6.15 mg of Cu, 70.0 mg of Fe, 65.0 mg of Zn, 47.0 mg of Mn, 0.135 mg of I, 0.115 mg of Co, and 0.115 mg of Mo.

**Table 2:** Effects of high-concentrate diet and thiamine supplementation treatment on the relative abundance of genes that encoded fiber-degrading enzymes.

Classification	Enzyme	Description	CON	HC	HCT	SEM	*p*-Value
Cellulose	CBM6	binding to cellulose	3.12 × 10^−4 a^	1.43 × 10^−4 b^	2.16 × 10^−4 ab^	4.49 × 10^−5^	0.026
	CBM16	binding to cellulose	4.01 × 10^−6 ab^	2.42 × 10^−7 b^	6.26 × 10^−6 a^	1.79 × 10^−6^	0.040
	GH3	β-glucosidase	1.95 × 10^−3 a^	9.30 × 10^−4 b^	2.03 × 10^−3 a^	3.13 × 10^−4^	0.022
	GH51	endoglucanase	4.89 × 10^−4 a^	1.88 × 10^−4 b^	3.03 × 10^−4 b^	8.84 × 10^−5^	0.038
	GH9	β-xylosidase	2.86 × 10^−4 b^	6.13 × 10^−4 a^	2.56 × 10^−4 b^	4.73 × 10^−5^	<0.001
	GH148	β-1,3-glucanase	1.92 × 10^−5 a^	4.38 × 10^−6 b^	6.67 × 10^−6 b^	1.99 × 10^−6^	0.001
	GH45	endoglucanase	8.11 × 10^−6^	1.76 × 10^−5^	7.69 × 10^−6^	8.20 × 10^−6^	0.442
	GH8	chitosanase	8.00 × 10^−5^	4.78 × 10^−5^	5.66 × 10^−5^	3.22 × 10^−5^	0.257
Hemicelluloses	GH2	β-galactosidase	1.73 × 10^−3 a^	6.68 × 10^−4 b^	1.26 × 10^−3 a^	1.65 × 10^−4^	0.002
	GT2	β-galactosidase	2.68 × 10^−3 a^	1.25 × 10^−3 b^	2.44 × 10^−3 a^	3.73 × 10^−4^	0.018
	GH35	β-galactosidase	2.10 × 10^−4 a^	7.05 × 10^−5 b^	1.72 × 10^−4 ab^	3.77 × 10^−5^	0.024
	GH5	chitosanase	6.09 × 10^−4 a^	3.05 × 10^−4 b^	3.80 × 10^−4 b^	9.06 × 10^−5^	0.035
	GH67	α-glucuronidase	1.01 × 10^−4 a^	2.79 × 10^−5 b^	6.92 × 10^−5 ab^	1.65 × 10^−5^	0.013
	CE1	Acetyl xyla esterase	7.45 × 10^−4 a^	3.19 × 10^−4 b^	5.37 × 10^−4 ab^	1.23 × 10^−4^	0.037
Total			2.39 × 10^−2 a^	1.27 × 10^−2 b^	2.03 × 10^−2 a^	2.40 × 10^−3^	0.009

^a–c^ Means within a row with different letters differed significantly (*p* < 0.05); SEM: Standard error of the mean. CON: Control diet; HC: High-concentrate diet; HCT: High-concentrate diet supplemented with thiamine. CBM: Carbohydrate-binding modules; GH: Glycoside hydrolase; GT: Glycosyl transferase; CE: Carbohydrate esterases.

**Table 3:** Effects of high-concentrate diet and thiamine supplementation treatment on the relative abundance of genes that encoded starch-degrading enzymes.

Enzyme	Description	CON	HC	HCT	SEM	*p*-Value
CBM41	starch-binding	4.38 × 10^−6 b^	2.35 × 10^−6 bc^	8.54 × 10^−6 a^	1.19 × 10^−6^	0.005
GH97	glucoamylase	7.08 × 10^−4 a^	1.75 × 10^−4 b^	4.94 × 10^−4 a^	1.02 × 10^−4^	0.006
GH133	amylo-α-1,6-glucosidase	4.73 × 10^−5 b^	1.60 × 10^−4 a^	1.98 × 10^−4 a^	1.92 × 10^−5^	0.001
GH13	α-amylase	1.07 × 10^−3 a^	5.32 × 10^−4 b^	1.19 × 10^−3 a^	1.59 × 10^−4^	0.018
GH31	α-glucosidase	2.96 × 10^−4 b^	5.43 × 10^−4 a^	6.83 × 10^−4 a^	7.57 × 10^−5^	0.006
GH77	amylomaltase	3.55 × 10^−4^	3.92 × 10^−4^	4.33 × 10^−4^	3.79 × 10^−5^	0.417
GH57	α-amylase	1.41 × 10^−4^	1.14 × 10^−4^	1.79 × 10^−4^	4.83 × 10^−5^	0.452
GH4	α-glucosidase	2.07 × 10^−5^	1.35 × 10^−5^	2.40 × 10^−5^	5.39 × 10^−6^	0.461
GH63	α-1,3-glucosidase	2.17 × 10^−5^	2.25 × 10^−5^	2.41 × 10^−5^	5.43 × 10^−6^	0.219
Total		5.45 × 10^−3 b^	4.23 × 10^−3 b^	6.776 × 10^−3 a^	6.12 × 10^−4^	0.018

a–c Means within a row with different letters differed significantly (*p* < 0.05); SEM: Standard error of the mean. CON: Control diet; HC: High-concentrate diet; HCT: High-concentrate diet supplemented with thiamine. CBM: Carbohydrate-binding modules; GH: Glycoside hydrolase; GT: Glycosyl transferase.

## Data Availability

Data is contained within the article or Supplementary Materials.

## Supplementary Materials

The following are available online at https://www.mdpi.com/2076-2607/9/3/632/s1. Table S1: Analysis of the metagenomic sequencing information of each sample; Table S2: CAZymes annotated in the CAZymes database by DIAMOND; Table S3: Effects of thiamine supplementation in a high starch diet on the dry matter intake and milk index; Table S4: Effects of thiamine supplementation in a high starch diet on the rumen fermentation index.
