# Supplementary material for: Metagenomic Insight: Dietary Thiamine Supplementation Promoted the Growth of Carbohydrate-Associated Microorganisms and Enzymes in the Rumen of Saanen Goats Fed High-Concentrate Diets
Source: Microorganisms. 2021 Mar 18;9(3):632. doi: 10.3390/microorganisms9030632 (PMC8003091; doi:10.3390/microorganisms9030632)
Supplement: Supplementary file 1 [file microorganisms-09-00632-s001.zip › Additional file/Table 1.docx]

Table S1 Analysis of the metagenomic sequencing information of each sample

| Sample | ORFs NO | Total length(Mbp) | Average length | GC(%) | N50 Len.(bp) | N90 Len.(bp) | Max len.(bp) |
| --- | --- | --- | --- | --- | --- | --- | --- |
| Con1 | 161,206 | 139.84 | 867.46 | 30.16 | 3,686 | 635 | 459,935 |
| Con2 | 170,007 | 123.84 | 728.41 | 36.86 | 2,563 | 637 | 455,374 |
| Con3 | 209,058 | 152.69 | 730.36 | 44.46 | 1,185 | 575 | 337,538 |
| HC1 | 163,338 | 121.19 | 741.98 | 50.16 | 1,429 | 623 | 159,785 |
| HC2 | 209,897 | 146.69 | 698.84 | 53.36 | 1,190 | 580 | 467,423 |
| HC3 | 160,340 | 114.53 | 714.3 | 41.9 | 1,851 | 616 | 562,073 |
| HCT1 | 226,098 | 156.6 | 692.61 | 39.9 | 1,186 | 581 | 324,321 |
| HCT2 | 232,078 | 153.16 | 659.96 | 50.56 | 2,028 | 635 | 259,586 |
| HCT3 | 236,096 | 159.47 | 675.45 | 49.05 | 1,694 | 606 | 267,531 |
