# Supplementary material for: Metagenomic Insight: Dietary Thiamine Supplementation Promoted the Growth of Carbohydrate-Associated Microorganisms and Enzymes in the Rumen of Saanen Goats Fed High-Concentrate Diets
Source: Microorganisms. 2021 Mar 18;9(3):632. doi: 10.3390/microorganisms9030632 (PMC8003091; doi:10.3390/microorganisms9030632)
Supplement: Supplementary file 1 [file microorganisms-09-00632-s001.zip › Additional file/Table 3-4.docx]

Table S3 Effects of thiamine supplementation in high starch diet on dry matter intake and milk index

| Item | CON | HC | THC | SEM | *P*-value |
| --- | --- | --- | --- | --- | --- |
| Dry matter intake (Kg/d) | 1.28^a^ | 1.03^b^ | 1.26^a^ | 0.09 | 0.012 |
| Milk yield (Kg/d) | 1.25^a^ | 0.93^b^ | 1.28^a^ | 0.07 | 0.024 |
| Milk component (%) | | | | | |
| Fat | 4.36^a^ | 3.52^c^ | 3.99^b^ | 0.11 | 0.008 |
| Protein | 4.17^a^ | 3.62^b^ | 4.20^a^ | 0.13 | 0.009 |
| Lactose | 4.57 | 4.53 | 4.58 | 0.09 | 0.209 |
| Yield (g/d) | | | | | |
| Fat | 54.5^a^ | 32.74^b^ | 51.01^a^ | 2.14 | 0.025 |
| Protein | 52.13^a^ | 33.67^b^ | 53.76^a^ | 1.59 | 0.009 |
| Lactose | 57.13^a^ | 42.13^b^ | 58.62^a^ | 2.31 | 0.018 |

Note: In the same row, values with no letter or the same letter superscripts mean no significant difference (*P*>0.05), while with different small letter superscripts mean significant difference (*P*<0.05). Same as below.

CON = control treatment; HC = high starch treatment; THC = high starch + thiamine treatment, thiamine added on a dry matter basis at 200 mg/Kg; 6 replicates per treatment (n = 6).

Table S4 Effect of thiamine supplementation in high starch diet on rumen fermentation index

| Item | CON | HC | THC | SEM | *P*-value |
| --- | --- | --- | --- | --- | --- |
| pH | 6.11^a^ | 5.42^b^ | 6.03^a^ | 0.11 | 0.003 |
| TVFA mmol/L | 65.22^c^ | 81.48^a^ | 72.98^b^ | 2.09 | 0.009 |
| Acetate (%TVFA) | 59.77^a^ | 33.53^b^ | 50.99^a^ | 2.08 | 0.007 |
| Propionate (%TVFA) | 24.16^c^ | 38.79^a^ | 28.88^b^ | 1.99 | 0.005 |
| Butyrate (%TVFA) | 13.08^c^ | 23.79^a^ | 16.55^b^ | 0.87 | 0.008 |
| Isobutyrate (%TVFA) | 1.72 | 1.67 | 1.75 | 0.12 | 0.078 |
| Valerate (%TVFA) | 0.64^c^ | 1.08^a^ | 0.88^b^ | 0.04 | 0.003 |
| Isovalerate (%TVFA) | 0.63^c^ | 1.13^a^ | 0.95^b^ | 0.07 | 0.006 |
| Acetate: Propionate | 2.47^a^ | 0.86^c^ | 1.77^b^ | 0.10 | 0.007 |
| Lactic acid mmol/L | 0.34^c^ | 0.71^a^ | 0.52^b^ | 0.04 | 0.003 |
| Pyruvate mmol/L | 0.21^c^ | 0.33^a^ | 0.26^b^ | 0.02 | 0.012 |
| PDH IU/L | 3.12^a^ | 1.03^c^ | 2.38^b^ | 0.11 | 0.009 |
| LPS Free LPS EU/mL (×10^3^) | 25.09^c^ | 55.87^a^ | 42.16^b^ | 3.08 | 0.011 |
| NH_3_-N mg/dL | 24.17^a^ | 11.28^c^ | 17.89^b^ | 1.21 | 0.006 |
| Thiamine μg/L | 7.15^a^ | 2.08^c^ | 4.02^b^ | 0.18 | 0.006 |

Note: In the same row, values with no letter or the same letter superscripts mean no significant difference (P>0.05), while with different small letter superscripts mean significant difference (P<0.05). Same as below.

CON = control treatment; HC = high starch treatment; THC = high starch + thiamine treatment, thiamine added on a dry matter basis at 200 mg/Kg; 6 replicates per treatment (n = 6)
